# Subinvolution of the placental site associated with focal retained products of conception and placenta accreta mimicking uterine arteriovenous malformation on CT and MRI: a lesson to be learned

**DOI:** 10.1590/0100-3984.2016.0131

**Published:** 2018

**Authors:** Laiz Laura Godoy, Ulysses S. Torres, Giuseppe D'Ippolito

**Affiliations:** 1 Hospital São Luiz, Grupo Fleury, São Paulo, SP, Brazil; 2 Hospital São Luiz, Grupo Fleury, e Escola Paulista de Medicina da Universidade Federal de São Paulo (EPM-Unifesp), São Paulo, SP, Brazil

**Figure 1:**
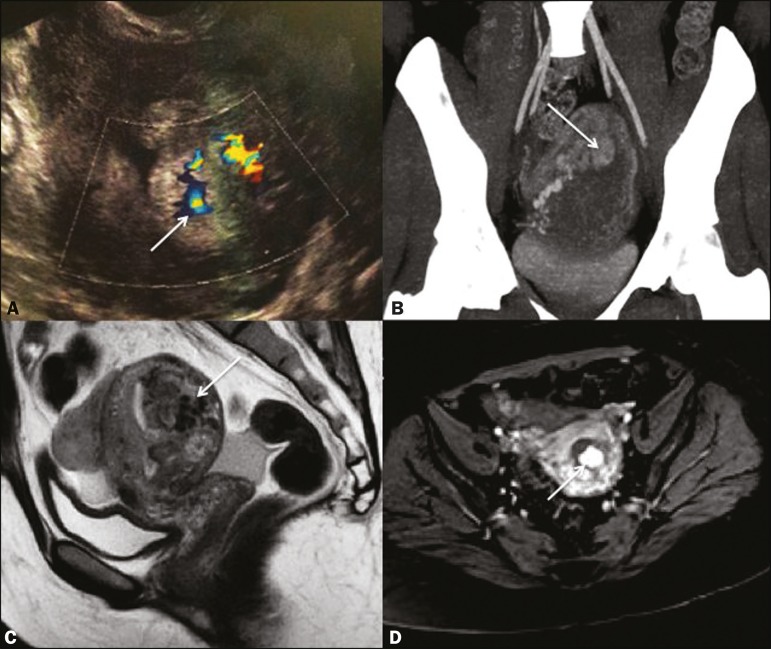
**A:** Transvaginal ultrasound showing a heterogeneous endometrial echo, with flow seen on the color Doppler study, especially in a grossly nodular formation in the basal region (arrow). **B:** Contrastenhanced CT scan with maximum intensity projection reconstruction identifying prominent myometrial and periuterine vessels in communication with the hypervascularized nodular area (arrow). C, **D:** Contrastenhanced MRI scans (sagittal T2-weighted and axial T1-weighted sequences, respectively) confirming the marked vascular dilatation, characterized by a flow void in the posterior uterine wall (arrow in C) and intense vascularization of the basal nodule (arrow in D).

Dear Editor,

Here, we report the case of a 36-year-old female patient (G5A4P1, undergoing cesarean section of twins) with a history of antiphospholipid antibody syndrome, gestational hypertension, and having undergone hysteroscopic procedures. On postpartum day 12, there was voluminous vaginal bleeding. Given the diagnostic hypothesis of retained products of conception (RPOC)—based on the finding of serum b-HCG values close to zero—we opted for clinical follow-up with ultrasound evaluations, which invariably showed a grossly nodular echogenic formation, measuring 2.2 cm at its greatest diameter and located near the basal endometrium, with internal vascular flow seen on color Doppler (Figure 1A). After approximately 60 days, the condition of the patient had not improved and the decision to perform curettage was therefore made. During the procedure, she bled profusely (500 mL) and became hypotensive. We did not identify any RPOC. Subsequent imaging of the pelvis, including a computed tomography (CT) scan (Figure 1B) and magnetic resonance imaging (MRI) scans (Figures 1C and 1D), confirmed the presence of a nodular formation near the basal endometrium, with intense contrast enhancement and communicating with a network of dilated and tortuous myometrial vessels. In correlation with the clinical data (bleeding that was difficult to resolve, significant worsening during surgical manipulation, and the absence of RPOC on curettage), the CT and MRI findings allowed the possibility of acquired arteriovenous malformation (AVM) to be considered^(1)^. Because conservative treatment was unsuccessful, we opted to perform a hysterectomy. The pathological diagnosis was RPOC in a focal area of placenta accreta with subinvolution of the placental site (SIPS).

In cases such as the one described here, the first pitfall is confusing the marked vascularization of RPOC with MAVs^(2)^. It should be borne in mind that RPOC occur much more frequently than do AVMs^(3)^, and it is therefore recommended that focal areas of uterine hypervascularity are simply reported as such, without necessarily relating them to AVMs^(4)^. In addition, an endometrial component of those focal changes favors a diagnosis of RPOC, whereas an unmistakably intramural component increases the suspicion of AVM^(2)^.

The second pitfall in cases such as this is the association with LIPS, an entity that can occur in the presence of RPOC (usually determined by focal accretions) or in isolation^(5,6)^. The prominent myometrial/periuterine vessels seen in patients with LIPS are indistinguishable from the findings in those with AVMs^(2)^. Therefore, because it is a rare diagnosis that is fundamentally histopathological^(5,7)^ and little discussed in the radiology literature, it is likely that LIPS also accounts for a portion of the cases of overdiagnosis^(4)^ and unconfirmed diagnosis of AVMs. Nevertheless, AVM is still rarer than in LIPS^(2,6)^.

In summary, when there is postpartum vaginal bleeding in a patient with normal b-HCG values and a finding of uterine hypervascular focal alteration, an endometrial component (RPOC) should first be excluded. When this differentiation is not clear, and especially when anomalous dilated myometrial vessels are detected in the adjacent areas, a diagnosis of LIPS accompanied by RPOC should be considered as a possible alternative to that of AVMs. The diagnosis of AVM can be confirmed by digital angiography, or the differentiation between the two diagnoses can be made through pathological study^(1,4)^.
